# Distinct Cardiac Transcriptional Profiles Defining Pregnancy and Exercise

**DOI:** 10.1371/journal.pone.0042297

**Published:** 2012-07-31

**Authors:** Eunhee Chung, Joseph Heimiller, Leslie A. Leinwand

**Affiliations:** 1 Department of Molecular, Cellular, and Developmental Biology, University of Colorado at Boulder, Boulder, Colorado, United States of America; 2 Biofrontiers Institute, University of Colorado at Boulder, Boulder, Colorado, United States of America; Johns Hopkins Univ. School of Medicine, United States of America

## Abstract

**Background:**

Although the hypertrophic responses of the heart to pregnancy and exercise are both considered to be physiological processes, they occur in quite different hormonal and temporal settings. In this study, we have compared the global transcriptional profiles of left ventricular tissues at various time points during the progression of hypertrophy in exercise and pregnancy.

**Methodology/Principal Findings:**

The following groups of female mice were analyzed: non-pregnant diestrus cycle sedentary control, mid-pregnant, late-pregnant, and immediate-postpartum, and animals subjected to 7 and 21 days of voluntary wheel running. Hierarchical clustering analysis shows that while mid-pregnancy and both exercise groups share the closest relationship and similar gene ontology categories, late pregnancy and immediate post-partum are quite different with high representation of secreted/extracellular matrix-related genes. Moreover, pathway-oriented ontological analysis shows that metabolism regulated by cytochrome P450 and chemokine pathways are the most significant signaling pathways regulated in late pregnancy and immediate-postpartum, respectively. Finally, increases in expression of components of the proteasome observed in both mid-pregnancy and immediate-postpartum also result in enhanced proteasome activity. Interestingly, the gene expression profiles did not correlate with the degree of cardiac hypertrophy observed in the animal groups, suggesting that distinct pathways are employed to achieve similar amounts of cardiac hypertrophy.

**Conclusions/Significance:**

Our results demonstrate that cardiac adaptation to the later stages of pregnancy is quite distinct from both mid-pregnancy and exercise. Furthermore, it is very dynamic since, by 12 hours post-partum, the heart has already initiated regression of cardiac growth, and 50 genes have changed expression significantly in the immediate-postpartum compared to late-pregnancy. Thus, pregnancy-induced cardiac hypertrophy is a more complex process than exercise-induced cardiac hypertrophy and our data suggest that the mechanisms underlying the two types of hypertrophy have limited overlap.

## Introduction

Cardiac hypertrophy is a prognostic indicator for heart disease and heart failure. Pathological cardiac hypertrophy in response to hypertension or mitral regurgitation results in concentric or eccentric cardiac hypertrophy, respectively. This remodeling is often associated with deleterious effects on cardiac function, and can progress to heart failure [1]. Unlike pathological cardiac hypertrophy, physiological hypertrophy, the process whereby neonatal hearts grow to adult size and athletes' hearts enlarge, is considered to be beneficial and this growth occurs while maintaining or improving cardiac function without inducing fibrosis or sarcomere disarray [2].

Pregnancy is another hypertrophic stimulus that is associated with a cardiac volume overload. In this respect, pregnancy-induced cardiac hypertrophy is somewhat similar to that induced by endurance exercise training. However, unlike exercise training, pregnancy is accompanied by significant changes in the hormonal milieu, and both the volume overload and increased heart rate are continuous rather than intermittent. Recently, we analyzed several signaling pathways at different time points including mid-pregnancy (MP) and late-pregnancy (LP) [3]. We showed that cardiac adaptation in MP shares some similarities with exercise training. For example, neither type of cardiac hypertrophy shows fibrosis. In addition, the Akt signaling pathway, which is important in exercise-induced cardiac hypertrophy [4], is also activated during pregnancy. However, pregnancy-induced cardiac hypertrophy also displays features distinct from exercise-induced hypertrophy. Unlike exercise, pregnancy is associated with short-term and transient systolic dysfunction indicated by decreased percent fractional shortening in LP [3], [5]. The hormonal milieu of pregnancy is also distinct from that of exercise, and several pieces of evidence suggest that these hormones impact cardiac hypertrophy during pregnancy. Progesterone, which is elevated during pregnancy, causes hypertrophy of neonatal rat ventricular myocytes [3]. Administration of estrogen to ovariectomized mice leads to decreases in cardiac Kv4.3 transcripts and increases in c-Src activity, mimicking what is seen during pregnancy [5]. For these reasons, the two settings are distinct, but their global transcriptional profiles have not been directly compared. Thus, the objective of this study was to test the hypothesis that pregnancy is accompanied by changes in cardiac gene expression that are distinct from those seen in exercise.

We examined the transcriptional profiles of hearts in response to pregnancy and exercise using Affymetrix microarrays (Mouse Genome 430 2.0 Array). A number of genes of interest in the various groups were validated experimentally through quantitative RT-PCR (qRT-PCR). We performed Gene Ontology (GO) analysis and pathway-oriented ontological analysis and found that while exercise and MP are very similar, they are distinct from LP and immediate-postpartum (0PP). Because GO analysis indicated a significant association of MP (but not EX) with genes involved in Ubl conjugation, we assessed proteasome activity at various time points, and found that it is significantly up-regulated in MP and 0PP, but down-regulated in 21EX. Altogether, our results demonstrate that pregnancy-induced and exercise-induced cardiac hypertrophy occur through molecular mechanisms that are similar when compared to MP but also distinct when considering LP and 0PP time points, suggesting that pregnancy-induced cardiac hypertrophy and regression follow a unique trajectory.

## Results and Discussion

In review articles [6], enlargement of the heart due to pregnancy and due to exercise are grouped together as “physiologic,” as opposed to pathologic. However, these two physiological pathways are, in fact, distinct due to many factors including the hormonal milieu of pregnancy and the continuous stimulus of pregnancy compared to the intermittent stimulus of exercise. Here, we report that pregnancy- and exercise-induced cardiac hypertrophy occur through molecular mechanisms that are similar at mid-pregnancy but very different at late-pregnancy and immediate-postpartum. To our knowledge, this is the first study to compare gene expression changes in two settings of physiological cardiac hypertrophy: exercise-induced cardiac hypertrophy vs. pregnancy-induced cardiac hypertrophy.

Cardiac hypertrophy, indicated by percent change following stimulus in left ventricular weight to tibial length (LV/TL) ratio compared to controls, was 14.0%, 18.6%, and 17.0% in MP, LP, and 0PP, respectively. The percent increase in LV/TL ratio in response to exercise was 22.1% and 28.5% in 7EX and 21EX, respectively. The detailed morphometric characteristics of female C57Bl/6 mice in response to pregnancy and exercise training are presented in **Table 1**. These time points were selected to identify patterns of gene expression that correspond to different phases of cardiac hypertrophy.

**Table 1:** Morphometric characteristics of female C57Bl/6 mice in response to pregnancy and voluntary wheel running.

	NP/Sed (n = 8)	MP (n = 7)	LP (n = 8)	0PP(n = 6)	7EX (n = 9)	21EX (n = 10)
BW (g)	20.47±0.36	25.58±1.22*	37.01±1.34*	24.66±0.69*	21.97±0.40*	23.34±0.53*
(mg)	69.71±1.74	81.73±2.79*	86.40±2.73*	83.68±2.30*	85.99±2.14*	90.85±1.72*
V/TL (mg/mm)	4.08±0.10	4.65±0.12*	4.84±0.13*	4.77±0.11*	4.98±0.10*	5.24±0.08*

Values expressed as mean ± standard error of mean (SEM). n = number of mice per group. Sed/NP, Sedentary/non-pregnant diestrus control; MP, mid-pregnancy; LP, late-pregnancy; 0PP, immediate- postpartum; 7EX, 7 days of voluntary wheel running; 21EX, 21 days of voluntary wheel running. BW, body weight; LV, left ventricular mass; TL, tibial length.

p≤0.05,

* significantly different from NP/Sed.

### Quantitative analysis shows two exercise groups are most closely related to mid-pregnant groups, but distinct from late-pregnant and immediate-postpartum groups

**Figure 1:**
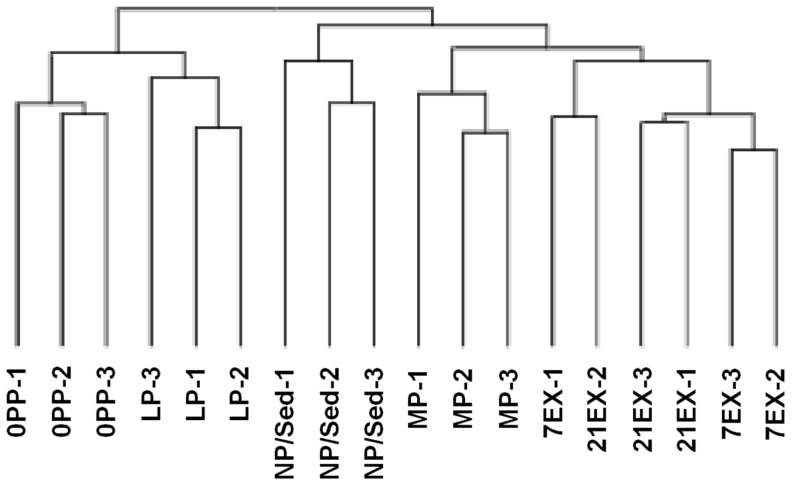
Hierarchical clustering demonstrates the MP gene expression is most closely related to the EX group. NP/Sed, virgin female mice at diestrus for non-pregnant sedentary controls; MP, mid-pregnancy; LP, late-pregnancy; 0PP, immediate-postpartum; 7EX, 7days of voluntary wheel running; 21EX, 21days of voluntary wheel running. Hierarchical clustering dendrogram generated using all probe sets with the ‘heatmap.2’ function in R.

**Figure 2:**
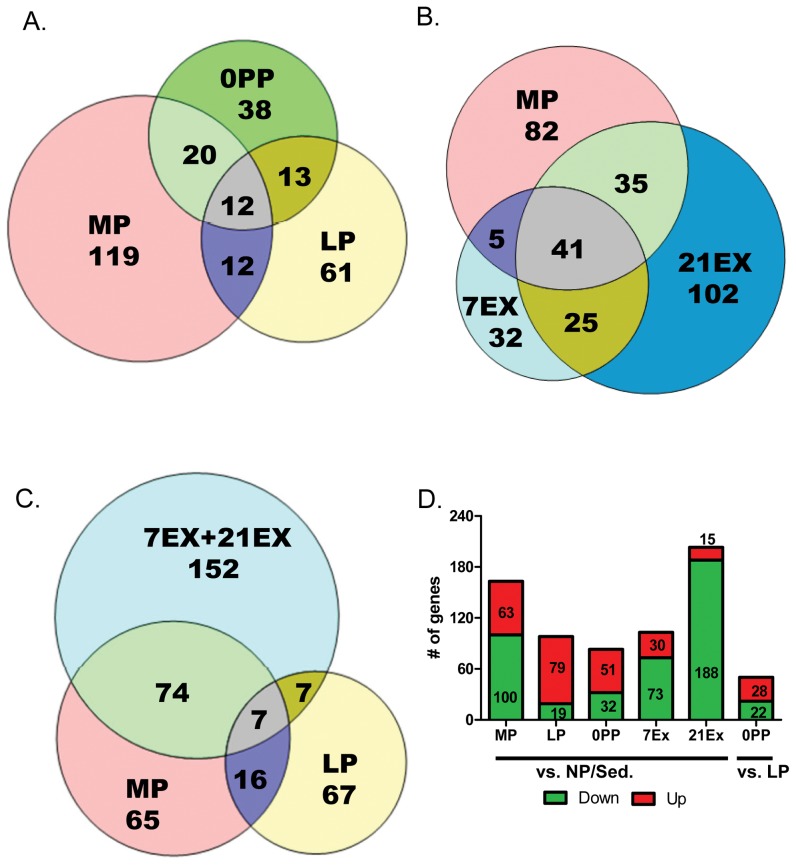
EX groups share gene regulation with MP. Venn diagrams of genes regulated A) during pregnancy, B) during MP, 7EX, and 21EX, C) during exercise, MP, and LP (all compared to NP/Sed. D) The number of genes changed compared to NP/Sed and comparison of 0PP to LP. Red represents genes up-regulated while green represents genes down-regulated.

In order to determine which groups had the most closely related expression profiles, hierarchical clustering of the microarrays was performed (**Figure 1**). As shown in **Figure 1**, the two exercise groups (7EX and 21EX) clustered together. One 7EX and one 21EX array clustered in the same branch, partially due to normal animal-to-animal variation in the temporal response to exercise. The MP group was most closely related to the EX groups. LP and 0PP each clustered together and were found to be distinct (in a separate branch) from NP/Sed, MP and EX. Next, we quantitatively assessed how different time points and stimuli affect gene expression profiles (**Figure 2**). First, we compared how many genes are differentially regulated during different time points in pregnancy. There are 163 genes in MP (63 up-regulated and 100 down-regulated), 98 genes in LP (79 up-regulated and 19 down-regulated), and 83 genes in 0PP (51 up-regulated and 32 down-regulated) differentially expressed compared to NP/Sed. There are only 12 genes shared among all three pregnancy groups (MP, LP, and 0PP). It is interesting to note that not many genes are shared between groups of different stages of pregnancy (**Figure 2A**: 24 genes between MP and LP, 25 genes between LP and 0PP, and 32 genes between MP and 0PP are shared). This result illustrates that each stage of pregnancy is associated with distinct programs of gene expression. In addition, the number of differentially expressed genes decrease as pregnancy progresses, suggesting a restoration of transcriptional levels comparable to those of NP/Sed. Further, we directly compared transcripts expressed in 0PP to LP (LP was the reference instead of NP/Sed because it is temporally much closer to 0PP), and surprisingly, 50 genes (28 genes up-regulated and 22 genes down-regulated) are differentially regulated within this short time period (the time between LP and 0PP is 1–2 days).

For exercise groups (**Figure 2B**), there are 103 genes differentially expressed in 7EX (30 up-regulated and 73 down-regulated), and 203 genes differentially expressed in 21EX (15 up-regulated and 188 down-regulated) compared to NP/Sed. While only 12 genes are shared among pregnancy groups, 66 genes are shared between 7EX and 21EX. Intriguingly, a large number of genes (76 genes) are shared between 21EX and MP although the degree of cardiac hypertrophy in 21EX (28.5%) is much greater than in MP (14.0%) compared to NP/Sed. When we compare EX (combined 7EX and 21EX) to MP (**Figure 2C**), 81 genes are shared between EX and MP. Among 81 genes, 80 genes are regulated in the same direction (6 genes are up-regulated and 74 genes are down-regulated). Only Zbtb16 is regulated in the opposite direction (down-regulated in EX and up-regulated in MP). Another interesting finding is that most genes are down-regulated in the EX groups (**Figure 2D**), and for the genes that do increase, the degree of up-regulation is lower in the EX group than in the pregnant groups (see **Table S1**). The entire list of genes altered across all groups is available as **Dataset S1**.

We compared our results to a publically available dataset on swimming-induced cardiac hypertrophy that had shown an almost identical degree of cardiac hypertrophy (29% in swimming vs. 28.5% in 21EX). The fold changes in response to one week of swimming are even lower, on average, than our voluntary wheel running group and yielded only 41 differentially expressed genes. Gene profiles from our voluntary wheel running exercise and the one-week swimming study suggest that neither form of exercise induces the large fold changes that are seen in pathological cardiac settings. For example, 865 genes are differentially regulated in isoproterenol-treated animals at a similar cutoff [7].

### Gene Ontology (GO) analysis supports quantitative analysis

**Figure 3:**
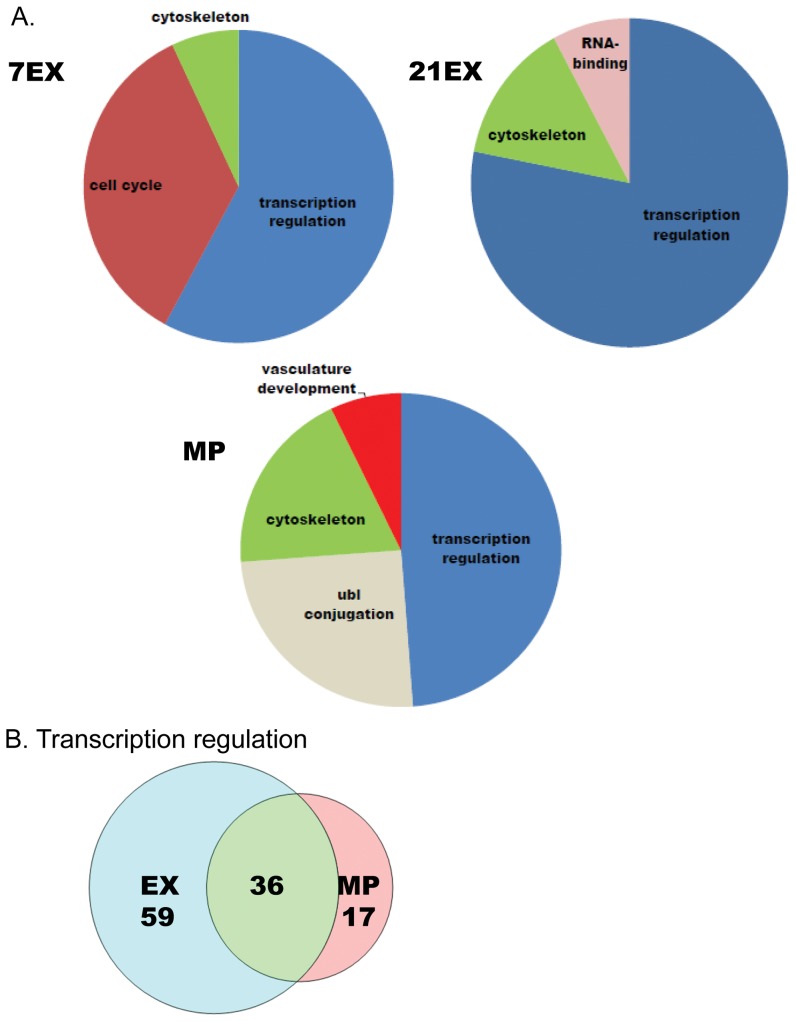
Gene ontology analysis of differentially expressed genes in MP and exercise groups. A) DAVID Gene Ontology analysis in 7EX, 21EX, and MP. B) The number of genes in the transcription regulation gene ontology category that are oppositely or similarly regulated in EX and MP.

We analyzed functionally related gene clusters of differentially expressed genes to provide an overview of the major processes regulating each group with DAVID (the Database for Annotation, Visualization, and Integrated Discovery). The ontology clusters are largely similar between MP and EX, including transcription regulation and cytoskeleton (**Figure 3A**), supporting the quantitative analyses shown in the Venn-diagram in **Figure 2**. We further asked how many genes are shared in the transcriptional regulation cluster between EX (7EX and 21EX) and MP. As shown in **Figure 3B**, 59 genes are uniquely regulated in EX, 17 genes are uniquely regulated in MP, and 36 genes are shared between EX and MP groups. Interestingly, except for Zbtb16 which is regulated oppositely depending on the stimulus (down-regulated in EX but up-regulated in MP), all other 35 shared genes are down-regulated in both EX and MP groups. Although alternative splicing is not included in pie chart due to the fact that GO clusters are less than 2% of the whole, several genes regulating alternative splicing are shared between 21EX and MP including Mbnl1, Sfrs3, Sfrs11, and Rbm25. Psip1, Fus, Sf3b2, Wbp11, Hspa8, Sf3b2 and Prpf40a are unique to 21EX, while Rbm5 is unique to MP. Mbnl1 is down-regulated in 21EX and MP and has been previously implicated in alternative splicing of genes related to muscle function, including cardiac troponin-T2 and Clcn1 (skeletal muscle chloride channel 1) [8]. Each group has distinct ontology clusters as well. Cell cycle is highly represented in 7EX, RNA-binding is highly represented in 21EX, and Ubl conjugation and vasculature development are uniquely regulated in MP (**Figure 3**).

**Figure 4:**
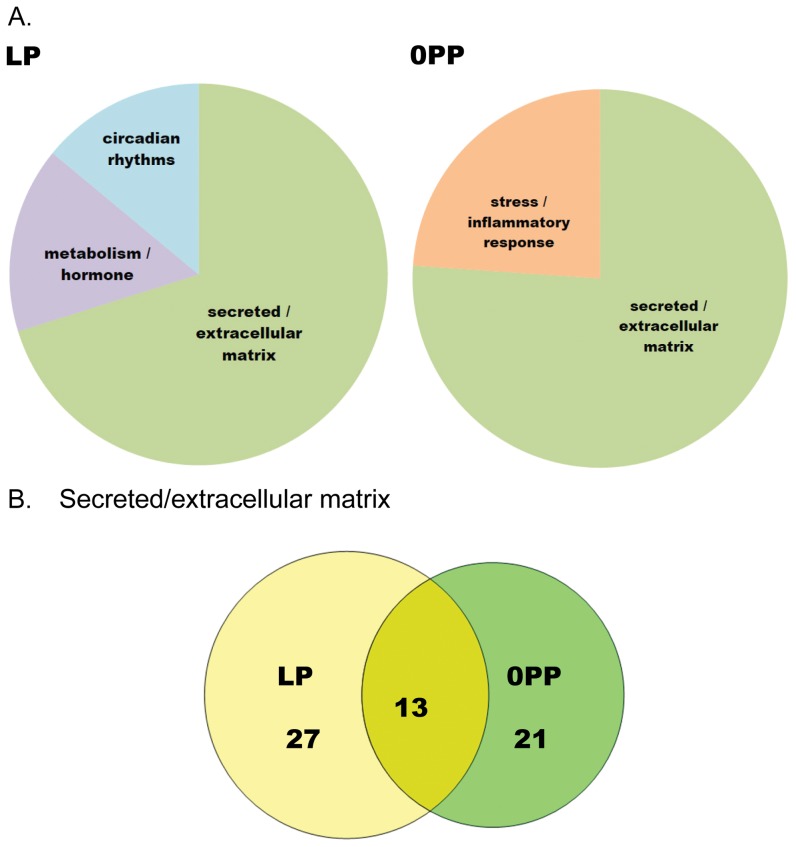
Gene ontology analysis of differentially expressed genes in LP and 0PP groups. A) DAVID Gene Ontology analysis in LP and 0PP. B) The number of secreted/extracellular matrix genes that are differently or similarly regulated in LP and 0PP.

While the MP and EX groups share many gene ontologies, the terms assigned to LP and 0PP are mostly distinct. Hormonal regulation/metabolism and biological rhythm are uniquely regulated in LP, while stress/inflammatory response is uniquely regulated in 0PP. The most statistically significant group for genes shared between LP and 0PP is secreted/extracellular matrix (**Figure 4A**). Because this is the most significant cluster, we compared the secreted/extracellular matrix-related genes between LP and 0PP groups (**Figure 4B**). The 13 genes that are shared between LP and 0PP are up-regulated, while 27 genes are uniquely regulated in LP. Surprisingly, 21 genes related to secreted/extracellular matrix are uniquely changed within 12 hours of parturition. The temporal gene expression profile during pregnancy is available as **Table S2**.

It has been suggested that fibrosis is the consequence of physical stress on the heart, such as increased afterload due to hypertension or increased volume overload that contributes to a reduction of systolic function [9]. We previously showed reduced systolic function during LP [3], but this decrease recovered to NP levels by 0PP (data not shown). Reduced systolic function due to pregnancy-induced volume overload may activate extracellular matrix genes during LP and 0PP. However, the activation of extracellular matrix genes does not induce fibrosis in the heart of pregnant mice (detailed histology in NP, MP, and LP is in [3]) and 0PP (data not shown). We have compared our data to a publically available volume overload microarray dataset, profiling male rats (GEO accession GSE12758). This comparison yielded very little overlap with our pregnancy data. Only 1 differentially expressed gene was found in common with MP (with the fold-change in the same direction), and there was no gene overlap in LP and 0PP. This suggests that pathological hypertrophy induced by volume overload has little in common with pregnancy-induced volume overload. Taken together, systolic dysfunction and activation of extracellular matrix genes during pregnancy are transient rather than persistent as seen in pathological cardiac hypertrophy [10].

### Validation of microarray data by qRT-PCR

**Figure 5:**
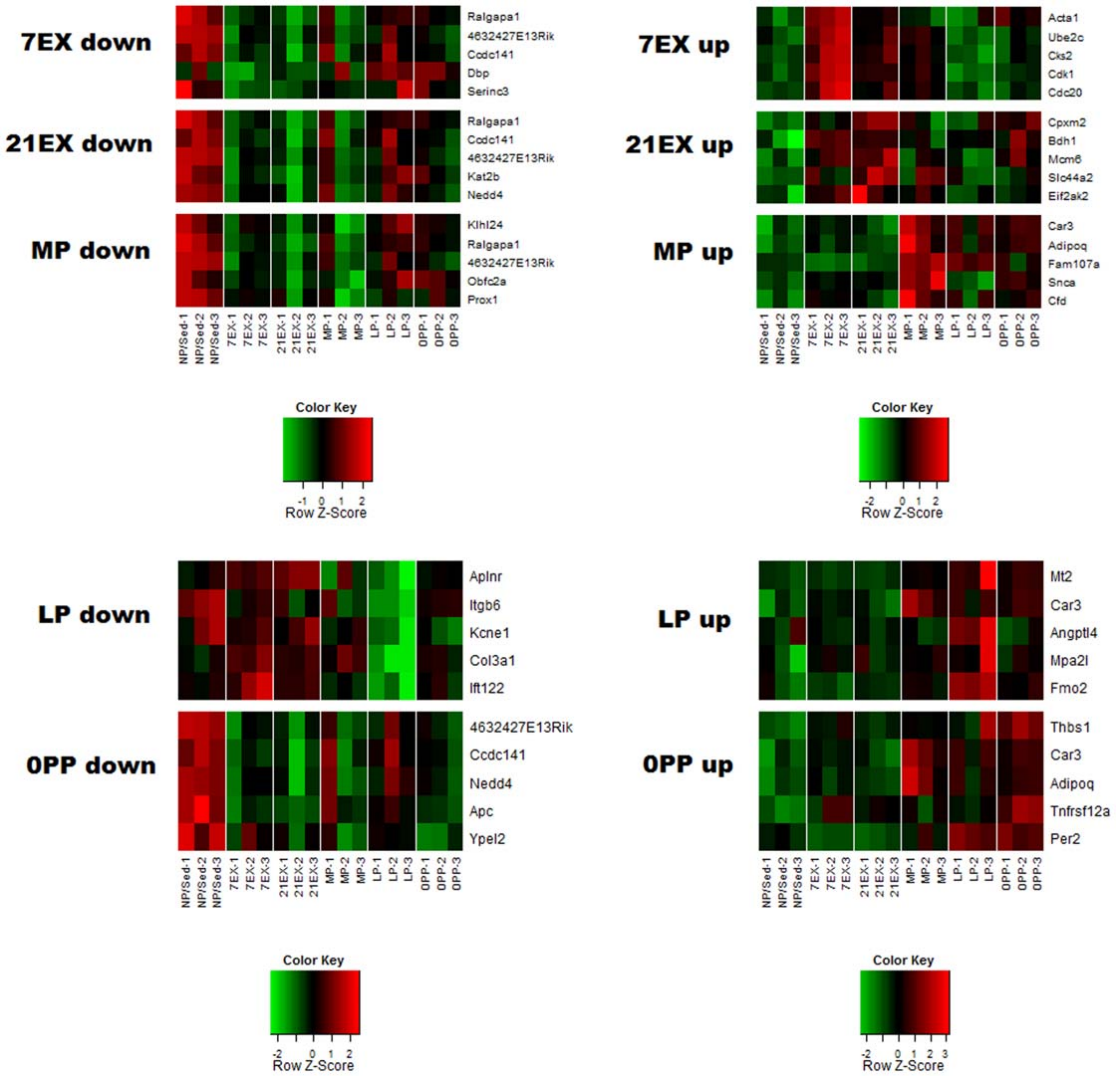
A heat map of the top differentially expressed genes in each group. The heat map of the top 5 genes, both up and down in fold-change, in each comparison group was created with the R heatmap.2 function. Z scores are computed separately for each probe set for the heat-map scale.

**Figure 6:**
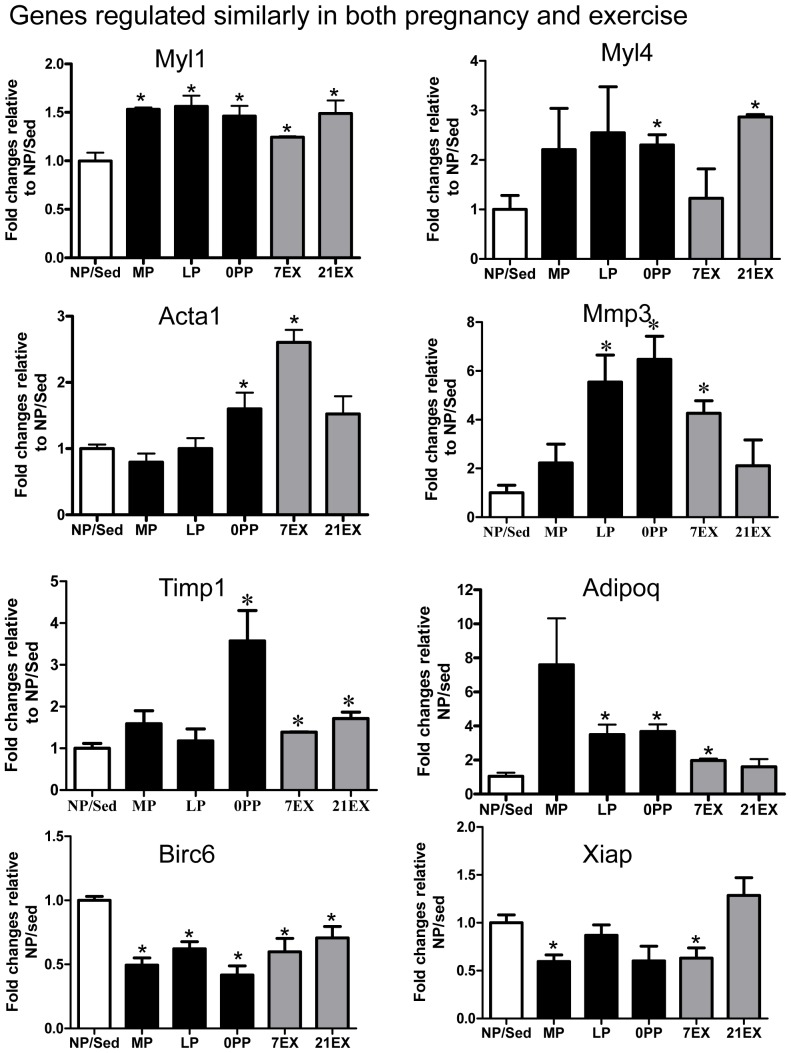
qRT-PCR of genes regulated similarly in pregnancy and exercise. Values are mean ± SEM expressed as fold change relative to NP/Sed. qRT-PCR was performed in duplicate with a minimum of 6 independent left ventricular samples per group. The levels of all mRNAs were normalized to 18S rRNA. *: p<0.05, significantly different from NP/Sed.

**Figure 7:**
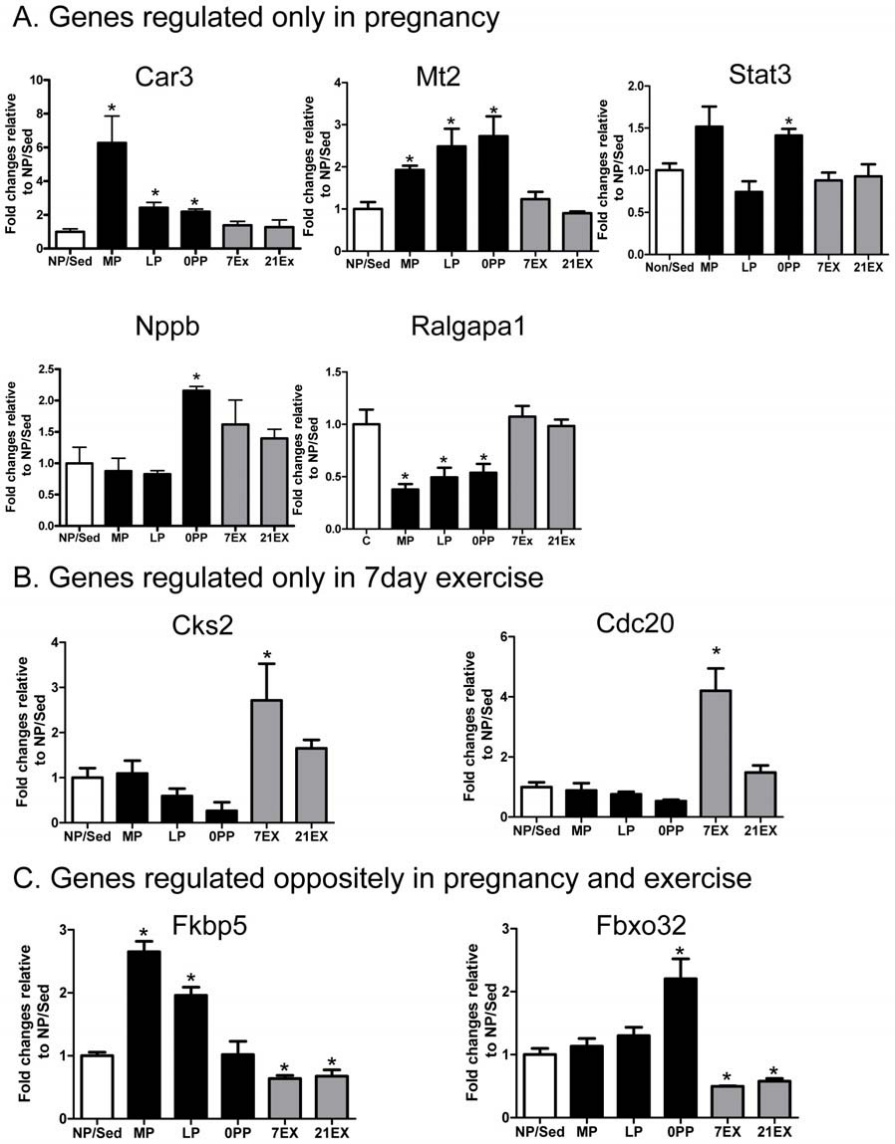
qRT-PCR validation of genes regulated in individual categories. A) Genes regulated only in pregnancy. B) Genes regulated only in the 7 day exercise group. C) Genes regulated oppositely in pregnancy and exercise. Values are mean ± SEM expressed as fold change relative to NP/Sed. qRT-PCR was performed in duplicate with a minimum of 6 independent left ventricular samples per group. The levels of all mRNAs were normalized to 18S rRNA. *: p<0.05, significantly different from NP/Sed.

Several molecules that are maximally regulated in each group are presented in **Figure 5** and a number of molecules that contribute to the significant GO groups were validated experimentally through quantitative RT-PCR (qRT-PCR). Verified genes were divided into: 1) genes regulated similarly in both pregnancy and exercise (**Figure 6**); 2) genes regulated only in pregnancy (**Figure 7A**); 3) genes regulated only in 7 days exercise (**Figure 7B**); and 4) genes regulated oppositely in pregnancy and exercise (**Figure 7C**).

### Genes regulated similarly in both pregnancy and exercise

We found Myl1 (fast skeletal myosin light chain 1) is significantly up-regulated in all pregnant and exercise groups, Myl4 (atrial myosin light chain 4) is significantly up-regulated in 0PP and 21EX, and Acta1 (α-skeletal actin) is significantly up-regulated in 0PP and 7EX. Previous studies show that Myl1 and Myl4 are significantly up-regulated in hearts from exercise-trained rats [11], [12]. Myl1 and Acta1 are significantly up-regulated in NRVMs treated with insulin-like growth factor 1 [12]. It has been demonstrated that the changes in expression of contractile protein isoforms are highly correlated with contractile properties. For example, upregulation of Myl4 protein in the ventricle has been associated with increased loaded shortening velocity and power output, as well as maximal force [13], [14]. An increase in Acta1 protein level is highly correlated with increased contractile function [15]. Taken together, genes encoding cytoskeletal proteins show changes in expression expected to enhance contractile properties in MP and EX.

Next, we validated Mmp3 (matrix metalloproteinase 3) and Timp1 (tissue inhibitors of metalloproteinase 1) that are important for extracellular matrix remodeling. The physiological condition of the extracellular matrix is maintained by a rigorously controlled balance between the synthesis and breakdown of its component proteins [16]. MMPs degrade collagen and other proteins present in the interstitial space, whereas TIMPs oppose the activity of MMPs. Dysregulation of MMPs and TIMPs is suggested as one of the mechanisms for the development of heart failure [17] due to adverse ventricular remodeling, leading to LV dilation and loss of contractile function. For example, plasma MMP3 levels are up-regulated soon after experimental acute myocardial infarction in animals and remains so for several days [18]. MMP3 protein levels are up-regulated in a decompensated heart failure model [19]. Timp1 increases in chronic pressure overloaded human hearts and its expression correlates with the degree of interstitial fibrosis [20]. However, Timp1 null mice show an increased hypertrophic response and adverse LV remodeling after myocardial infarction [21], and Timp1 overexpression significantly reduces hypertrophic growth of cardiomyocytes and prevents cardiac dilation during acute left ventricle pressure overload [20]. Together, these suggest that fine tuning of MMPs and TIMPs is important for cardiac remodeling. We found that Mmp3 is significantly up-regulated in LP, 0PP, and 7EX, while Timp1 is significantly up-regulated in 0PP, 7EX, and 21EX. Thus, unlike pathological cardiac hypertrophy, the Mmp3/Timp1 ratio is well maintained in both pregnancy- and exercise-induced cardiac hypertrophy. In addition, Adipoq (adiponectin) has been shown to protect hearts from myocardial ischemia-reperfusion injury [22], and we found that Adipoq is up-regulated in pregnancy and 7EX (see **Figure 6**). Previously, Kong et al. [23] demonstrated that genes for cytoskeletal and extracellular cellar matrix proteins are involved in both pathological hypertrophy and physiological hypertrophy induced by 6 weeks of treadmill exercise training. In agreement with this previous study [23], several genes related to cytoskeletal and extracellular matrix proteins are also highly regulated in both pregnant and EX groups (**Figure 6**), but the mode of regulation may be more favorable in EX and pregnancy, such as maintaining the ratio of MMPs to TIMP.

Apoptosis is generally associated with pathological hypertrophy and heart failure [24], while exercise training has been shown to attenuate apoptosis [25]. Birc6 (baculoviral IAP repeat-containing protein 6) and Xiap (X-linked inhibitor of apoptosis) promote cell survival by inhibiting apoptosis. They are known to be E3 ligases to catalyze the ubiquitination of caspase-3 and caspase-9 [26]. Previous studies show that Birc6 significantly decreases in idiopathic dilated cardiomyopathy [27]. Xiap either significantly increases [28] or does not change [29] in response to exercise training, and significantly increases in heart failure [29]. Surprisingly, these two anti-apoptotic molecules are among the top molecules that are down-regulated during exercise and pregnancy. The microarray data show that Bclaf1 (Bcl2-associated transcriptional factor 1), which is pro-apoptotic, is also significantly down-regulated. Thus, we determined apoptotic activity by monitoring the rate of cleavage of a fluorogenic caspase-3 specific substrate from whole heart homogenates from each group. Caspase-3 activity was not detectable except in the LP group, but the level of caspase-3 activity in LP was negligible (0.2457±0.014/mg protein). We used thymus as a positive control for this assay and the caspase3 activity of thymus was 22.64±3.04/mg protein. No increase in apoptosis during pregnancy and exercise is probably due to the continuous and balanced regulation of anti- and pro-apoptotic genes during pregnancy and exercise. Thus, the hearts are protected from fibrosis.

### Genes regulated only in pregnancy

Car3 (carbonic anhydrase III), Mt2 (metallothionein 2), Stat3 (signal transducer and activator of transcription 3), Nppb (natriuretic peptide precursor B), and Ralgapa1 (Ral GTPase activating protein, alpha subunit 1) are uniquely regulated in pregnancy but not altered in EX groups. Car3 is up-regulated in pregnancy (**Figure 7A**). Car3 catalyzes the reversible hydration of carbon dioxide and binds to the Na^+^-H^+^ exchanger (NHE1), thereby participating in acid-base balance [30]. It has been shown that Car3 is more highly expressed in female hearts than male hearts [31], perhaps due to regulation of NHE1 by estradiol [32]. In addition, over-expression of Car3 protects hearts from oxidative stress [33]. Therefore, increased Car3 during pregnancy may be protective. Mt2 is also one of the most highly up-regulated molecules unique to pregnancy (**Figure 7A**). It has been shown to protect hearts from oxidative injury [34]. Transgenic over-expression of Mt2 in mice has protective effects on acute and chronic oxidative stress conditions, such as treatment with doxorubicin and ischemia-reperfusion [34] and confers resistance to diabetes-induced cardiomyopathy [35]. In addition, Stat3, which is involved in the protection of the heart from oxidative stress, is significantly up-regulated in 0PP (**Figure 7A**). A previous study has shown that deletion of Stat3 in female mice leads to the development of postpartum cardiomyopathy by increasing reactive oxygen species [36]. Taken together, during pregnancy, it is very important to protect the heart from the oxidative stress by up-regulating genes that have antioxidant properties, such as Car3, Mt2, and Stat3.

Nppb (Natriuretic peptide precursor B), also known as BNP, is often up-regulated with pathological cardiac hypertrophy. In addition, women with heart disease have higher plasma BNP levels during pregnancy and after delivery compared to women without heart disease [37]. On the other hand, Nppb has been shown to exert anti-hypertrophic and anti-fibrogenic effects on the heart, and knockout mice deficient in the BNP receptor, guanylyl cyclase A (GC-A), undergo cardiac hypertrophy and develop extensive interstitial fibrosis [38]. Thus, a transient abrupt increase in Nppb in 0PP may protect a heart that is undergoing dramatic changes during parturition (**Figure 7A**). In addition, Ralgapa1, a molecule that is involved in Gα12/13 signaling, is significantly down-regulated in MP, LP, and 0PP (**Figure 7A**), whereas Gα12/13 signaling is significantly up-regulated in pathological cardiac hypertrophy [39].

### Genes regulated only in 7 day exercise groups

It is generally accepted that the heart is post-mitotic, but this has been challenged by the postulation that adult cardiomyocytes have the potential to proliferate in response to exercise training [40]. Our gene expression profiling show that genes related to cell cycle, including Ccna2, Ccnb2, Cdc20, Cdk1, Cep55, Cks2, Mcm5, Mki67, Smc2, Top2a, and Ube2c are all up-regulated in 7EX. In contrast, genes related to inhibitors of cell cycle progression, such as Cdkn1b and Kat2b are significantly down-regulated in 7EX. Our results agree with a previous study where cellular proliferation was the most over-represented molecular function after swim training in mice [7]. Among these genes, we validated Cks2 (CDC28 protein kinase regulatory subunit 2) and Cdc20 (cell division cycle 20 homolog), and these are up-regulated only in 7EX (**Figure 7B**). Thus, we and the other group [7] support the recent work done by Bostrom et al. [40], which suggests that adult cardiomyocytes have the potential to proliferate in response to exercise-training. However, since we profiled the whole left ventricle and not isolated cardiomyocytes, we cannot rule out that these are actually changes in the gene expression of other cell types in the heart.

### Genes regulated oppositely in pregnancy and exercise

Fkbp5 (FK506 binding protein 5) and Fbxo32 (atrogin1) are regulated by both pregnancy and exercise, but the directions are different between groups. Fkbp5 and Fbxo32 are up-regulated in pregnant groups, while down-regulated in exercise groups (**Figure 7C**). Fkbp5 is involved in the modulation of steroid receptor function, including progesterone, androgen, and glucocorticoid receptors [41], and has been shown to be more highly expressed in female hearts compared to male hearts [31], [42]. Previous studies demonstrated that Fkbp5 is the most strongly up-regulated target gene in progesterone signaling [43], and we previously demonstrated that serum progesterone levels are highest in MP and maintained until near term [3], which correlates with up-regulation of FKBP5 in MP and LP. In contrast, Fkbp5 is significantly down-regulated in both 7EX and 21EX. Fbxo32 (atrogin-1), one of the E3 ligases, which has been shown to have an important role in muscle atrophy [44], is significantly up-regulated in 0PP but down-regulated in both 7EX and 21EX, suggesting that hearts initiate cardiac regression when hypertrophic stimuli ceases. This validation of the microarray data by qRT-PCR demonstrates the quality of the microarrays and provides justification for proceeding with pathway-oriented ontological analyses.

### Pathway-oriented ontological analysis

**Figure 8:**
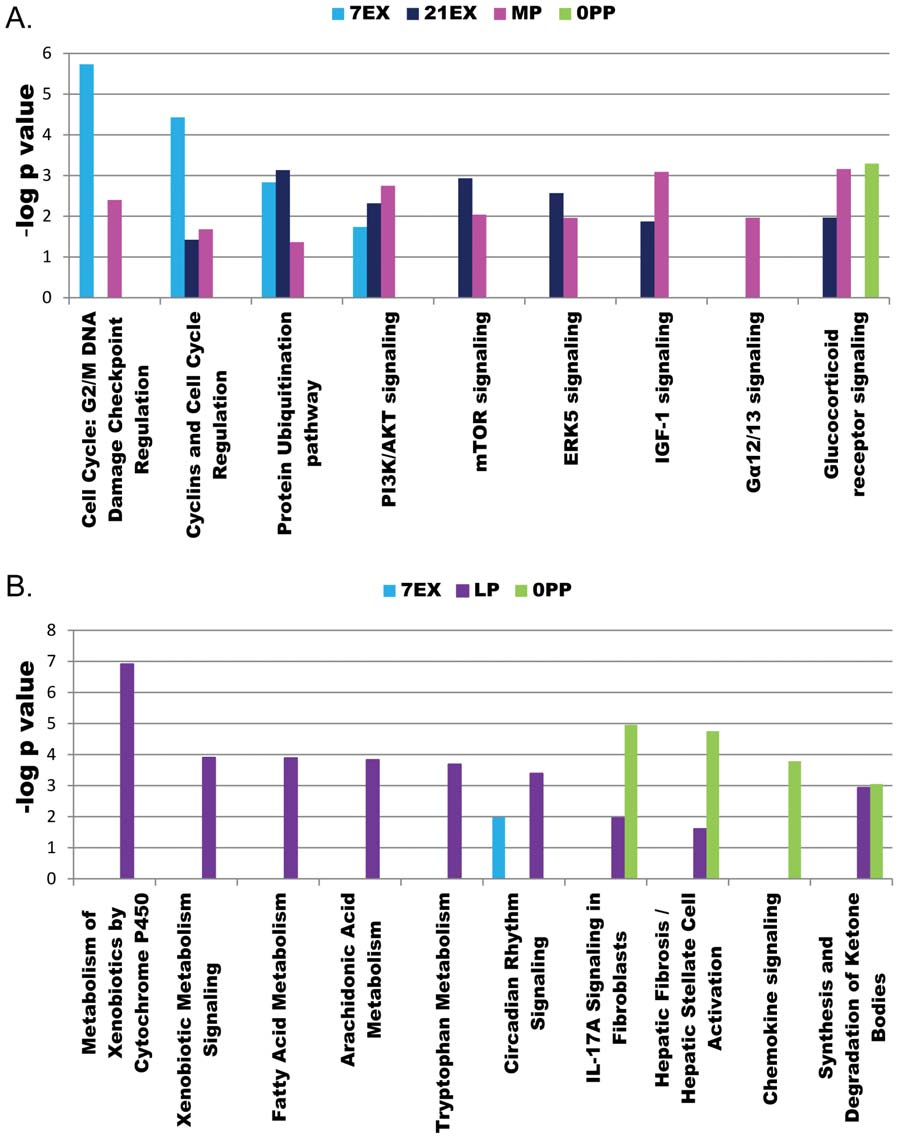
Pathway-oriented ontological analysis demonstrates similarities between EX and MP but distinct from LP and 0PP. A) Statistically significant pathways largely shared between EX and MP. B) Statistically significant pathways in LP and 0PP.

Since a large number of genes are shared between 21EX and MP and between LP and 0PP, we further analyzed the data using the canonical pathways analysis tool within IPA to predict which pathways are responsible for cardiac remodeling at different stages of pregnancy and exercise. Once again, many canonical pathways are shared between EX and MP including cell cycle regulation, protein ubiquitination pathway, PI3K/Akt signaling, mTOR signaling, ERK5 signaling, and IGF-1 signaling, but none of these pathways is involved in LP and 0PP (**Figure 8**). Of these pathways, PI3K/Akt signaling, mTOR signaling, and IGF-1 signaling are well-known to be involved in for exercise-induced cardiac hypertrophy and cell survival [45]. In addition, we previously demonstrated that the phosphorylation status of Akt and its downstream targets, including GSK3β, mTOR, and p70S6 kinase, are significantly increased in MP, but return to NP levels in LP [3]. The predicted pathway analyses support our previous findings that cardiac adaptation during MP is similar to the response to exercise. In addition, ERK5 signaling is regulated in both 21EX and MP. ERK5 signaling is related to lengthening of cardiomyocytes that induces eccentric cardiac hypertrophy [46], whereas ERK1/2 activation produces concentric cardiac hypertrophy [47]. We previously demonstrated that the phosphorylation status of ERK1/2 is significantly increased in MP, and our current study shows that ERK5 signaling is highly regulated in both MP and 21EX. Taken together, we can speculate that physiological hypertrophy, as defined by an increases in wall thickness proportional to increased chamber diameter, in response to pregnancy and exercise is possibly modulated by both ERK5 and ERK1/2 signaling.

In contrast, we found Gα12/13 signaling, one of the important pathways responsible for maladaptive cardiac hypertrophy leading to heart failure [39], is only regulated in MP. However, the signaling molecules involved in Gα12/13 signaling (Rasa1, Rhoa, and Ralgapa1) are significantly down-regulated, demonstrating that physiological hypertrophy is distinct from pathological cardiac hypertrophy with respect to these signaling molecules. In agreement with GO analysis, cell cycle regulation is most highly regulated in 7EX and moderately in 21EX and MP. Glucocorticoid receptor signaling is a significant pathway in MP, 21EX and 0PP. In addition, MP shows some evidence of thyroid-related regulation with the differentially expression of Nr4a1 (nuclear receptor subfamily 4, group A, member1), Thrsp (thyroid hormone responsive), and Cxcl1 (chemokine C-X-C motif ligand 1) [48], while 21EX group shows differentially regulation of Tef (thyrotrophic embryonic factor) and Trip11 (thyroid hormone receptor interactor 11) (**Dataset S1**).

The top five predicted pathways for LP include metabolism of xenobiotics by cytochrome P450, xenobiotic metabolism signaling, fatty acid metabolism, arachidonic acid metabolism, and tryptophan metabolism. Interestingly, cytochrome P450 enzymes (Cyp) are involved in all top five predicted pathways, and Cyp1a1, Cyp1b1, Cyp2e1, and Cyp4b1 are up-regulated in LP, suggesting the importance of Cyp in LP (**Dataset S1**). Cyp is a family of mono-oxygenases that are able to metabolize arachidonic acid [49] and steroid hormones [50]. Cyp1a1, Cyp1b1, Cyp2e1, and Cyp4b1 are constitutively expressed in the heart [51], [52], [53], and their levels are altered in response to pathological stimuli [52]. For example, Cyp1a1 and Cyp1b1 are significantly increased by isoproterenol-induced cardiac hypertrophy [52] and 24 h after doxorubicin administration [49]. Cyp2e1 is either significantly decreased by isoproterenol-induced cardiac hypertrophy [52] or remains unchanged in response to doxorubicin-induced cardiotoxicity [49]. In addition, there is a strong correlation between arachidonic acid metabolites and the pathogenesis of cardiac hypertrophy. Arachidonic acid can be metabolized by Cyp to epoxyeicosatrienoic acids (EETs) that have cardioprotective effects, while hydroxyeicosatetraenoic acids (HETEs) that are known to be a detrimental in many cardiovascular diseases [54]. Isoproterenol-induced cardiac hypertrophy has been shown to disturb this balance with increased formation of the cardiotoxic 20-HETE and decreased formation of the cardioprotective EETs. Estradiol can be metabolized by Cyp1a1 and cyp1b1 to 2-/4-Hydroxyestradiol and these metabolites are more potent than estradiol to prevent cardiac fibrosis [55]. Considering the effects of metabolites of steroid hormones and arachidonic acid on cardiac disease, further research into the potential impact on pregnancy-induced cardiac hypertrophy is warranted.

The pathways predicted by Ingenuity Pathway Analysis in 0PP are fibrosis and chemokine signaling. It has been demonstrated that estradiol inhibits collagen synthesis and cardiac fibroblast growth, and combined with progesterone enhances the inhibitory effects of estradiol [56]. Unlike pathological stimuli that significantly up-regulate collagen isoforms [7], Col3a1 and Col15a1 are significantly down-regulated in LP, when estradiol levels are maximal, but these genes return to NP levels when estradiol levels return to NP level in 0PP. Previously, we showed that both estradiol and progesterone change in a time-dependent manner during pregnancy. For example, serum estradiol levels are significantly increased at MP and maximal at LP, whereas serum progesterone levels gradually increase and peak at MP and these levels are maintained through LP [3]. The levels of estradiol and progesterone in 0PP return to NP levels. Thus, within 12 hours of parturition, the hormonal milieu is dramatically changed, and these changes may activate many genes associated with fibrosis and chemokine signaling. In addition, estradiol reduces the adhesion of activated monocytes to the endothelium by inhibiting the expression of cell adhesion molecules, such as VCAM1 [57]. However, Vcam1 is significantly up-regulated in 0PP where the estradiol levels have returned to NP levels, suggesting that decreased estradiol may activate extracellular matrix remodeling and chemokine signaling.

### Proteasome activity

**Figure 9:**
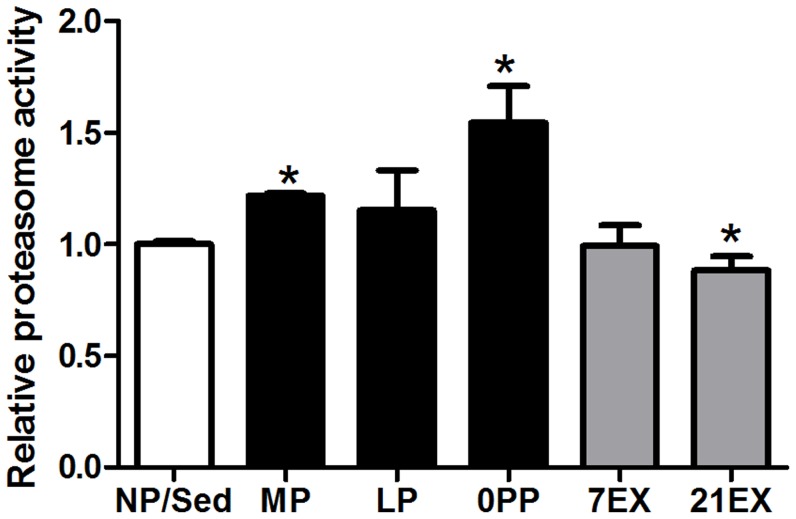
Proteasome activity is oppositely regulated in EX and pregnant group. Values are mean ± SEM expressed as fold change relative to NP/Sed. 4–6 hearts per each group were used. *: p≤0.05, significantly different from NP.

The ubiquitin proteasome pathway is a major pathway regulating protein turnover [58]. We measured proteasome activity for following reasons: 1) Ubl conjugation and protein ubiquitination pathways, are a highly regulated GO group (**Figure 3**) and pathway oriented ontological analysis (**Figure 8**), respectively and 2) Fbxo32 (atrogin1) is significantly altered but in opposite directions during pregnancy and exercise (**Figure 7C**). Interestingly, proteasome activity is significantly increased in MP and 0PP, but significantly decreased in 21EX (**Figure 9**). It has been shown that proteasome activity plays important roles in skeletal muscle loss [59] and cardiac atrophy [60]. Counterintuitive to its role in atrophy, proteasome activity is significantly increased in pressure-overload induced cardiac hypertrophy, diabetes-induced cardiomyopathy, and doxorubincin-induced cardiac toxicity [61]. Furthermore, previous studies demonstrated the beneficial effects of proteasome inhibition on pathological hypertrophy. Proteasome inhibitors block development of pathological hypertrophy without impairing contractile function [62]. Thus, the role of proteasome activity in pregnancy-induced cardiac hypertrophy warrants further study.

In summary, our data demonstrate that substantial expression changes take place in genes related to transcription regulation and cytoskeleton (EX and MP), myocardial vasculature (MP), extracellular remodeling (LP and 0PP), and stress/inflammatory response (0PP). Although the duration of stimuli is similar between LP (18–19 days) and 21EX, percent changes in LV/TL compared to NP/Sed are much greater in 21EX (28.5%) than in LP (18.6%). We can speculate that hypertrophic signaling may have plateaued at MP by activating PI3K/Akt, mTOR, and IGF signaling (**Figure 8**), but other processes, such as extracellular matrix remodeling, mainly occur during LP and 0PP. This study provides ample evidence that, while both pregnancy and exercise are considered to be physiological stimuli of cardiac hypertrophy, they employ mechanistically distinct processes during adaptation and thus should not be thought of interchangeably.

## Materials and Methods

### Ethics Statement

All of the animals were handled and euthanized under the guidelines of University of Colorado Animal Use and Care Committee, consistent with regulations for vertebrate animal research outlined by the National Institutes of Health. The title of protocol, “Mediators of Cardiac Adaptation (#1002.08)” was approved on April 1, 2010 by the University of Colorado at Boulder Animal Care and Use Committee.

### Animals

Three- to four-month old female C57Bl/6 mice were used for both the pregnancy and exercise studies. Mice used for the pregnancy group were described previously [3]. Briefly, pregnancy groups were composed of 11 days of gestation (MP) and 18–19 days of gestation (LP), and within 12 hours of parturition (0PP). These time points allowed us to identify distinct patterns of gene expression throughout pregnancy. For exercise groups, mice were subjected to voluntary wheel running for either 7 days (7EX) or 21 days (21EX) as described previously [63]. We used virgin female mice at diestrus for non-pregnant sedentary controls (NP/Sed). Animals were housed in a temperature- and light-controlled room with food and water available ad libitum.

At a given time point, mice were weighed and then sacrificed by cervical dislocation after inhalation of isoflurane. Hearts were rapidly excised and washed in PBS to allow blood to be pumped out of the cardiac chambers and coronary vessels. The hearts were trimmed of connective tissue, vascular tissue, atria, and right ventricle. The left ventricles were blotted dry and weighed. The left ventricle was immediately frozen in liquid nitrogen, and stored at −80°C for total RNA isolation.

### Total RNA isolation for microarray analysis and Quantitative real time PCR

Total RNA was isolated from frozen left ventricular tissues using TRI Reagent (MRC, Inc.: Cincinnati, OH) and further purified with RNeasy Mini kits (Qiagen; Valencia, CA) according to the manufacturer's instructions. Gene expression profiling was done at the Molecular, Cellular, and Developmental Biology Microarray Core facility of the University of Colorado-Boulder. Briefly, equal amounts of total RNA were pooled from two to three hearts for each chip to decrease animal-to-animal variability. Biotin-labeled amplified RNA was fragmented and hybridized onto the microarrays (Mouse Genome 430 2.0 Arrays) according to the Affymetrix protocol.

A number of genes in the various groups were validated experimentally through quantitative RT-PCR (qRT-PCR). 2 µg of total RNA was reverse transcribed with the High-Capacity cDNA Reverse Transcription Kit (Applied Biosystems, CA, USA) with random primers according to the manufacturer's instructions. qRT-PCR was done either by SYBR Green or TaqMan gene expression assay (Applied Biosystems, CA, USA) with an Applied Biosystems 7500 Real-Time PCR system. 18s rRNA was used for normalization of candidate genes. Primers for 18s, Nppb, and Acta1 were listed previously [3], [64]. Additional primers are listed in **Protocol S1**.

### Gene expression profiling and bioinformatic analyses

The gene expression data were deposited in the Gene Expression Omnibus (GEO) database (http://www.ncbi.nlm.nih.gov/projects/geo/) and can be retrieved with GEO accession number GSE36330. Microarray analysis was performed with the R statistical environment version 2.12.2 (http://www.r-project.org) using the Bioconductor package [65]. The RMA method with default options was used for normalization, background correction and summarization across all microarrays [66]. Eighteen microarrays were analyzed in all (n = 3 per each group). Hierarchical clustering was performed using the R heatmap.2 function to cluster the microarrays by expression level similarity, using the Manhattan distance metric and all probe sets (**Figure 1**). Local false discovery rates for each probe set were computed across microarray groups with the Cyber-T function bayesT using the PPDE (Posterior Probability of Differential Expression) analysis. The Cyber-T method partly compensates for lack of replication by adjusting variance using similar expression-level probe sets [67] and has been shown to outperform other common methods using spiked-in datasets [68], [69]. Chip quality was assessed using the affyPLM module's image function in R. Probe sets were considered differentially expressed across conditions if the comparison Cyber-T PPDE local false discovery rate was ≥0.95 and the fold-change difference was ≥75% (log-2 fold-change absolute value ≥0.807).

Gene Ontology (GO) analysis was performed with the DAVID functional annotation online analysis (https://david.abcc.ncifcrf.gov/) [70] using as a background only probesets that were called as ‘Present’ on at least one of the microarrays. Pie charts were generated using DAVID Functional Annotation Clustering output. Each pie chart slice represents a DAVID ontology cluster (labeled by the most common ontology theme of the ontology groups in the cluster). Each slice percentage represents the percentage of genes in that ontology cluster out of all the possible genes in the ontology cluster. If a gene was assigned to more than one significant cluster, it was only included in the cluster with the highest DAVID enrichment score (some high-level GO groups were ignored because of their ambiguity). Ontology clusters that represented less than 2% of the whole were not included.

Ingenuity Pathway Analysis (IPA) version 7.6 (www.ingenuity.com) was used to create the bar chart of unique and common pathway-oriented ontological analysis between the comparison groups. For each group we used the set of differentially expressed probe sets with the same significance thresholds as described above. A P value≤0.05 was used as the cutoff for significance for the pathways, calculated with a right-tailed Fisher's exact test (this is the standard significance threshold used in IPA, shown as a −log(P value)≥1.3). Not all pathways were used in the bar chart.

For the microarray analysis of the publicly-available dataset from swimming-exercised mice, we used the E-MTAB-27 dataset from ArrayExpress using 8-week old FVB mice. This dataset also used left ventricular tissue from female mice (http://cardiogenomics.med.harvard.edu/groups/proj1/pages/swim_home.html). The week zero microarrays using non-exercised mouse samples were used as a control (6 arrays) and week one swimming-exercised mice microarrays were used in the swimming group (3 arrays). The one week time point was chosen because it was closest in percent heart increase to our 21-day exercised mice (29% in swimming vs. 28.5% in our 21-day voluntary wheel-exercised mice). A 50% fold-change (a lower fold-change was used to account for the modest changes in this dataset) and 0.05 p-value (Student's t-test) was selected as the cutoff for significance for the swimming dataset, which yielded 41 genes.

The volume overload comparison was performed with the GEO GSE12758 dataset. This experiment profiled male rats using a shunt (versus a sham control) to induce volume overload. We used only the left ventricle data (6 arrays in all). A 75% fold-change cutoff was selected as the cutoff for significance for this dataset, which yielded 38 genes.

### Caspase-3 activity and Proteasome activity assay

Caspase-3 activity was measured by monitoring the rate of cleavage of fluorogenic caspase-3 specific substracte (Acetyl-AspGluValAsp-AMC: Calbiochem) as described previously [71]. Proteasome activity assays were measured using fluorogenic peptide substrates as described previously [72]. The proteasome activity was measured with 35 µg of total protein extracts of left ventricle with Suc-LLVY-AMC (Boston Biochem) as the substrate. Assay was carried out over 1 hour and activity was determined by calculating the slope of the linear portion of the reaction. All measurements were performed in duplicate and 4–6 animals per group were used.

### Statistical analysis

Validated genes by qRT-PCR were expressed as mean ± standard error of mean (SEM). Statistical significance was tested with Student's t-test to compare the differences between NP/Sed and each treatment group (MP, LP, 0PP, 7EX, and 21EX). P<0.05 was regarded as significant between groups.

## Supporting Information

Dataset S1 **Gene expression statistics by gene across all comparisons.** This excel file contains the list of genes that are significant across the groups. Column C, Expression level graphs contain log-2 fold change to show up- and down-regulation of each group compared to NP/Sed. Doublets indicate microarray with qRT-PCR validation. Other fold change columns (E, G, I, K, M, O, Q, S, U, W, Y, AA, AC, AE, and AG) are ratios (not log2). The ‘best’ vs ‘average’ columns: The average statistic columns are blank if there is only one probe set for the gene. If there are multiple probe sets for the same gene the ‘best’ columns show the statistic for the ‘best’ probe set in terms of False Discovery Rate value and the ‘average’ columns show the average of all probe sets. *, A gene was considered differentially expressed across conditions if one of gene's probe set across the comparison Cyber-T PPDE local false discovery rate was ≥0.95 and the fold-change difference was ≥75% (log-2 fold-change absolute value ≥0.807). File may be slow to load due to embedded graphics.
(XLSX)

Protocol S1 **Primers for Quantitative RT-PCR.**
(DOCX)

Table S1 **Top molecules regulated by each group compared to NP/Sed.**
(DOCX)

Table S2 **Temporal gene expression pattern during pregnancy.**
(DOCX)
