# Supplementary material for: Distinct Cardiac Transcriptional Profiles Defining Pregnancy and Exercise
Source: PLoS One. 2012 Jul 31;7(7):e42297. doi: 10.1371/journal.pone.0042297 (PMC3409173; doi:10.1371/journal.pone.0042297)
Supplement: Protocol S1 — Primers for Quantitative RT-PCR. (DOCX) [file pone.0042297.s002.docx]

Mmp3, Fwd 5’- ACATGGAGACTTTGTCCCTTTTG-3’, Rev 5’- TTGGCTGAGTGGTAGAGTCCC-3’; Timp1, Fwd 5’- CTTGGTTCCCTGGCGTACTC-3’, Rev 5’- ACCTGATCCGTCCACAAACAG-3’; Birc6, Fwd 5’- ACAGATTGTCTTACCTCTTGCCC-3’, Rev 5’- GCCACGAAGTGAAGGTCTCC-3’; Xiap, Fwd 5’- AGTTGTCATGCGGCAATAGATAG-3’, Rev 5’- CTGTCAGGGGCAAAAGGATTT-3’; Stat3, Fwd 5’- TCCTGGCACCTTGGATTGAGA-3’, Rev 5’- AGGAATCGGCTATATTGCTGGT-3’; Ralgapa1, Fwd 5’- GGGAAGGTGTTCGTCTCTTCT-3’, Rev 5’- TCCAGGGATCAAGCATGAAAAC-3’; Cks2, Fwd 5’- TACTTCGATGAGCACTACGAGT-3’, Rev 5’- AAGAGAAGAATATGCGGTTCTGG-3’; Cdc20, Fwd 5’- GTTCGTGTTCGAGAGCGATTT-3’, Rev 5’- ATGGGTGCGTCCAGTTGAAG-3’; Fkbp5, Fwd 5’- TGAGGGCACCAGTAACAATGG-3’, Rev 5’- CAACATCCCTTTGTAGTGGACAT-3’; Fbxo32, Fwd 5’- CTTTCAACAGACTGGACTTCTCGA-3’ Rev 5’- CAGCTCCAACAGCCTTACTACGT-3’. The TaqMan Probes used in this study includes Adipoq (Mm00456425_m1*), Car3 (Mm00483016_m1), Myl1 (Mm00659043_m1), Myl4 (Mm00440378_m1*), and Mt2 (Mm00809556_S1).
