# Supplementary material for: Distinct Cardiac Transcriptional Profiles Defining Pregnancy and Exercise
Source: PLoS One. 2012 Jul 31;7(7):e42297. doi: 10.1371/journal.pone.0042297 (PMC3409173; doi:10.1371/journal.pone.0042297)
Supplement: Table S1 — Top molecules regulated by each group compared to NP/Sed. (DOCX) [file pone.0042297.s003.docx]

**Table S1. Top molecules regulated by each group compared to NP/Sed**

|  | 7EX | 21EX | MP | LP | 0PP |
| --- | --- | --- | --- | --- | --- |
| Up | Acta1 (2.67) | Cpxm2^+^ (2.38) | Car3^+^ (6.59) | Mt2 (3.92) | Thbs1^+^ (4.04) |
|  | Ube2c (2.59) | Bdh1^+^ (2.15) | Adipoq (5.51) | Car3^+^ (3.43) | Car3^+^ (3.95) |
|  | Cks2^+^ (2.57) | Mcm6^+^ (2.03) | Fam107a^+^ (4.19) | Angptl4^+^ (3.32) | Adipoq (3.03) |
|  | Cdk1 (2.49) | Slc44a2^+^ (1.96) | Snca^+^ (4.15) | Mpa2l^+^ (3.00) | Tnfrsf12a^+^ (2.64) |
|  | Cdc20^+^ (2.46) | Eif2ak2^+^ (1.96) | Cfd (4.00) | Fmo2^+^ (3.01) | Per2^+^ (2.63) |
|  | Bdh1^+^ (2.44) | Isg15 (1.91) | Fos (3.13) | Fkbp5^+^ (2.91) | Ccl2 (2.58) |
|  | Lcn2 (2.37) | Limd2^+^ (1.90) | Fam46C (3.10) | Adh1^+^ (2.89) | Timp1 (2.52) |
|  | Hist1h2ae (2.29) | Ifit1 (1.90) | Nr41a1 (3.02) | Per2^+^ (2.89) | Nppb (2.39) |
|  | Aspm^+^ (2.20) | Arntl^+^ (1.89) | Alas2 (2.76) | Hmgcs2^+^ (2.85) | Ccl7 (2.37) |
|  | Top2a^+^ (2.16) | Ifit3 (1.85) | Cdkn1A^+^ ( 2.62) | Fam107a^+^ (2.85) | Hmgcs2^+^ (2.26) |
| Down | Ralgapa1^+^ (0.38) | Ralgapa1^+^ (0.30) | Klhl24^+^ (0.39) | Aplnr^+^ (0.42) | 4632427E13Rik(0.41) |
|  | 4632427E13Rik (0.39) | Ccdc141(0.33) | Ralgapa1^+^ (0.39) | Itgb6^+^ (0.45) | Ccdc141^+^ (0.44) |
|  | Ccdc141(0.39) | 4632427E13Rik^+^ (0.36) | 4632427E13Rik(0.41) | Kcne1^+^ (0.49) | Nedd4^+^ (0.46) |
|  | Ddp^+^ (0.40) | Kat2b^+^ (0.37) | Obfc2a^+^ (0.43) | Col3a1^+^ (0.493) | Apc^+^ (0.49) |
|  | Serinc3^+^ (0.406) | Nedd4^+^ (0.38) | Prox1^+^ (0.448) | Ift122^+^ (0.50) | Ypel2^+^ (0.493) |
|  | Kat2b^+^ (0.45) | Zfp800(0.38) | Atrx^+^ (0.46) | Clasp1^+^ (0.52) | Rad23b^+^ (0.50) |
|  | Zfp800^+^ (0.45) | Rbm25^+^ (0.39) | Nedd4^+^ (0.47) | Arntl^+^ (0.53) | Clasp1^+^ (0.50) |
|  | Nedd4^+^ (0.45) (0.46) | Birc6^+^ (0.40) | Mbnl1^+^ (0.47) | Atp5a1^+^ (0.54) | Gbf1^+^ (0.51) |
|  | 6820431F20Rik | Ttn (0.40) | Rad23b^+^ (0.47) | Prox1^+^ (0.54) | Bbx^+^ (0.51) |
|  | Kitlg^+^ (0.46) | Xiap^+^ (0.41) | Nr3c1^+^ (0.47) | Auts2^+^ (0.54) | Ralgapa1^+^ (0.51) |

Average fold changes were indicated in parenthesis; ^+^ indicates multiple probe sets
