# Supplementary material for: Distinct Cardiac Transcriptional Profiles Defining Pregnancy and Exercise
Source: PLoS One. 2012 Jul 31;7(7):e42297. doi: 10.1371/journal.pone.0042297 (PMC3409173; doi:10.1371/journal.pone.0042297)
Supplement: Table S2 — Temporal gene expression pattern during pregnancy. (DOCX) [file pone.0042297.s004.docx]

**Table S2. Temporal gene expression pattern during pregnancy**

|  | Ontology categories: associated genes |
| --- | --- |
| Up throughout pregnancy | **chemokine/immune response**: Ccl6, Ccl9, Cfd, Ifi205, Lcn2, Lyve1, Mt2, Mpa2l, Ppbp; **extracellular/secreted**: Adamts1, Adipoq, Thbs1, Stbd1 ; **metabolism**: Bdh1, Hecw2, Adh1, Fmo2, Ugt1a1; **transcription regulation**: Cebpd, Chd6, Fos; **contraction**: Myl1; **stress response**: Car3; **growth**: Nov; **membrane/signaling**: Ms4a6d**; steroid receptor**: Fkbp5 |
| Down throughout pregnancy | **regulation of transcription**: Bptf, Eny2, Zc3h4, Zfp800, Sox4, Hif1a, Ilf3, Mef2a; **immune**:, Jam2, Kitl; **extracellular/secreted**: Angpt1, Cdh4, Cdh13; **ubiquitin-mediated proteolysis**: Nedd4, Rad23b, Tbl1x; **GTPase**: Ralgapa1, Gbf1, Clasp1; **Tight Junction**: Magi3; **phosphoprotein**: Stk39; **Wnt signaling**: Apc; **Cell morphogenesis**: Prox1; **nucleus**: Ypel2; **unknown functio**n: Secisbp2l, D330013E07Rik, C030011G24Rik, 4632427E13Rik, 6820431F20Rik |
| Up in MP and then down | **immune response**: Ifi27l1, Gbp3, Irf7, Rsad2, Oasl2; **ubiquitin**: Isg15, Usp18, Ube2l6; **nuclear receptor**: Nr4a1; **blood**: Hba-a1, Ahsp; **anion exchange**: Slc4a1; **unknown function**: 6030422H21Rik |
| Down in MP and then up | **cytoskeletal**: Gmfb, Slmap; **RNA processing**: Syncrip, Fyttd1; **ubiquitin-mediated proteolysis**: Zfand5; **DNA repair**: Obfc2a; **apoptosis**: Serinc3; **GTPase**: Gdi2; **phosphoprotein**: D4Wsu53e; **unknown function**: C230091D08Rik |
| Only up in 0PP | **Cytoskeleton**: Acta1, Synpo21; **Biological rhythm**: Egr3; **stress response**: Hspa1b; **anti-fibrotic/extracellular**: Nppb, Itgb1bp3 |
| Only down in 0PP | 0 genes |
| Up in LP and 0PP | **extracellular matrix**: Mmp3, Prg4; **biological rhythm**: Per2; **ketogenesis**: Hmgcs2; **Ca ^2+^ binding**: Calr3; **smooth muscle contraction**: Ptgds |
| Down in LP and 0PP | 0 genes |
| Up in MP and LP, then down in 0PP | **steroid hormone transport**: Slc10a6 |
| Down in MP and LP then up in 0PP | 0 genes |
